# Quantum Collision Models: A Beginner Guide

**DOI:** 10.3390/e24091258

**Published:** 2022-09-07

**Authors:** Stefano Cusumano

**Affiliations:** International Centre for Theory of Quantum Technologies, University of Gdansk, 80-308 Gdańsk, Poland; stefano.cusumano@ug.edu.pl

**Keywords:** collision model, tutorial, open quantum systems, master equation

## Abstract

In recent years, quantum collision models, sometimes dubbed repeated interaction models, have gained much attention due to their simplicity and their capacity to convey ideas without resorting to technical complications typical of many approaches and techniques used in the field of open quantum systems. In this tutorial, we show how to use these models, highlighting their strengths and some technical subtleties often overlooked in the literature. We do this by deriving the Markovian master equation and comparing the standard collisional derivation with the standard microscopic one. We then use the collision model to derive the master equation of a two-level system interacting with either a bosonic or fermionic bath to give the reader a flavour of the real use of the model.

## 1. Introduction

Any physics student that starts studying quantum mechanics is first taught about closed systems, i.e., systems that can be fully described by a Hamiltonian operator and a ket. Going further in the study of quantum physics, the student realizes that Hamiltonian dynamics can only describe a small, and often unrealistic, fraction of quantum systems. In reality, most physical systems are not closed, but rather open: by this it is meant that they are surrounded by an environment, whose presence influences the system in a way that cannot be described by a Hamiltonian operator alone. As a consequence, one must find a way to account for the presence of the environment in the dynamical equations describing the system.

The interaction with the environment is responsible for many physical phenomena, such as spontaneous emission and frequency shift [1,2], the emergence of the classical world [3,4] and the description of thermodynamic phenomena [5].

The description of open quantum systems is still nowadays a central problem in physics [6]. While first approaches to the problem relied on phenomenological equations [7,8,9], a critical turning point was arrived at with the derivation of the Gorini–Kossakowski–Sudarshan–Lindblad (GKSL) master equation [10,11]: starting from a Hamiltonian description of the system and the environment, the environmental degrees of freedom are traced away under certain assumptions, leading to a master equation which is guaranteed to be completely positive and trace-preserving, thus having full physical meaning.

Other approaches to the study of open quantum systems were conceived later, such as quantum trajectories [12,13], Monte Carlo method [14] and quantum jumps [15] and input–output formalism relying on stochastic calculus [16,17,18].

While the aforementioned methods are generally very effective, they often rely on complicated mathematics, hiding the physics behind the model. On the other hand, in recent years, growing attention has been devoted to quantum collision models. While such methods are related to the aforementioned ones [19,20,21,22,23], they rely on a much simpler mathematical description, allowing one to better focus on the physics of the model.

In collision models, the dynamics is discretized by depicting the environment as a collection of quantum systems, typically dubbed ancillas, which interact one by one with the system through a unitary operation. These ancillary quantum systems are then traced away, so that one is left with the state of the system alone. In this way, the dynamics is effectively divided into time slices, giving a step-wise description of the dynamics of the system. Another advantage of this approach is that, thanks to the discreteness of the environment, it is also possible to study how its state changes during the interaction with *S*.

Collision models were first conceived in [24], and since then they have been used to describe a large number of physical situations: Markovian [25] and non-Markovian [26,27,28,29,30] dynamics, the role of correlations between system and environment [31,32,33,34], global vs local master equations [35], heat exchange and work extraction in quantum thermodynamics [36,37,38,39,40,41,42], quantum optics [43,44,45], cascaded systems [46,47,48,49,50] and many others [51].

This tutorial goes as follows. In Section 2, we review the derivation of the Markovian master equation in the GKSL form step by step in both the microscopic and collisional approach. This will allow us to highlight the differences between the two approaches and to stress the importance of the approximations made, which can become useful also for beginners in the field of open quantum systems. Then, in Section 3 we analyze a couple of examples, namely a two-level system interacting with a bosonic bath and a spin bath. Finally, in Section 4 we conclude the paper, summarizing what has been done and making some final remarks.

## 2. Basic Collision Model: The Markovian GKSL Master Equation

When modeling an Open Quantum System (OQS), one has to keep in mind first the physical situation from which the dynamics is originated: namely, one always deals with a closed system, S+E, made by two subsystems, one being the system *S* we wish to describe through our dynamical equations, and the other being the environment E. The joint evolution of these two subsystems taken together can usually be considered closed, i.e., described by the usual Schrödinger equation corresponding to the total Hamiltonian. However, as the environment is typically made out of a huge number of degrees of freedom, it is impossible to keep track of the full dynamics of the joint system, and thus the usual approach is to trace away the degrees of freedom of the environment in order to obtain a master equation describing system *S* alone.

**Figure 1:**
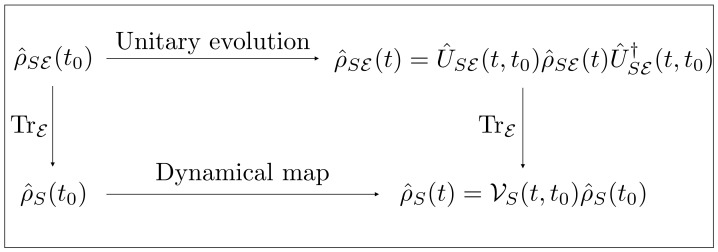
Diagram summarizing the procedure to describe open system dynamics: one starts with a joint state ${\hat{\rho }}_{SE}$ of system and environment, then one can either cause the state to evolve through unitary dynamics and then trace away the environmental degrees of freedom, or, conversely, first trace away the environment and then apply to system *S* the dynamical map describing the open dynamics.

In order to obtain meaningful dynamical equations, the tracing of the environmental degrees of freedom must be carried out carefully, making appropriate approximations on the joint dynamics of S+E (see Figure 1). This gives rise to a plethora of different master equations and approaches, as already mentioned in the introduction to this tutorial. The most important and simple equation when dealing with open quantum systems is the Markovian GKSL master equation, which can be used to describe many physical situations, and is usually used as the starting point in the analysis of more complicated situations.

Thus, in order to better understand collision models, and to highlight their main strengths, weaknesses and subtleties, it is useful to derive the Markovian GKSL master equation using a collision model, and to compare this with the usual microscopic derivation, which can be found in standard books such as [6].

In the following, we will describe the various steps that lead to the Markovian GKSL master equation using both methods, starting from the initial description of the joint S+E system, then turning to the dynamical description in both derivations, and finally comparing how the environmental degrees of freedom are traced away.

### 2.1. Modeling the System

In both the microscopic and collisional derivation, the system *S* is described through a Hamiltonian ${\hat{H}}_{S}$. In the microscopic approach, the environment E is described as a set of modes with free Hamiltonian ${\hat{H}}_{E}$, interacting with *S* through the interaction Hamiltonian ${\hat{H}}_{SE}$.

On the other hand, in the collisional approach the environment E is described as a collection of quantum systems $\left\{E_{i}\right\}$, typically called ancillas and described by the Hamiltonian ${\hat{H}}_{E_{i}}$, each of which interacts piecewise with *S* through the interaction Hamiltonian ${\hat{H}}_{SE_{i}}$.

Thus, in the microscopic approach, the joint S+E system is described through the total Hamiltonian:

(1) $$\begin{array}{c} {\hat{H}}_{mic}={\hat{H}}_{S}+{\hat{H}}_{E}+{\hat{H}}_{SE}, \end{array}$$

while in the collisional description we have:

(2) $$\begin{array}{c} {\hat{H}}_{col}={\hat{H}}_{S}+\sum_{i}{\hat{H}}_{E_{i}}+{\hat{H}}_{SE_{i}}. \end{array}$$

**Figure 2:**
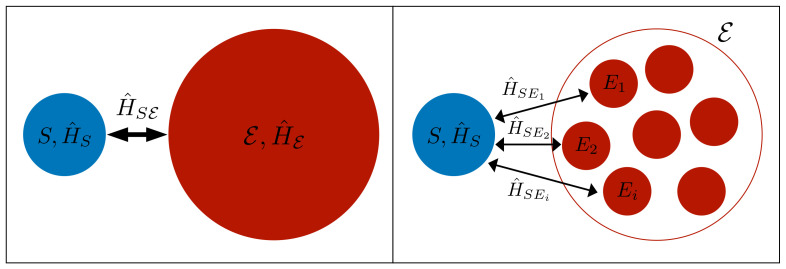
A pictorial representation of the microscopic (**left**) and collision (**right**) model. In the microscopic approach, the environment is represented as a uniform and continuous set of modes, while in the collision model it is represented as a discrete collection of quantum systems.

Here, we can already see a first difference between the microscopic and the collisional approach: while in the first case the environment is represented as a continuous “jelly-like” set of degrees of freedom, in the collision model the environment is discretized into ancillary quantum systems interacting piecewise with the system. This is pictorially represented in Figure 2, where it is also highlighted that in the collision model the interaction between the system and the environment is represented via the interaction of the system with each single ancilla.

Moreover, it is normally assumed the the system and the environment are initially in a product state such as:

(3) $$\begin{array}{c} {\hat{\rho }}_{SE}\left(0\right)={\hat{\rho }}_{S}\left(0\right)⊗{\hat{\rho }}_{E} \end{array}$$

where ${\hat{\rho }}_{E}$ is the state of the environment, most often taken to be a thermal state, though in principle any choice is possible.

In the collision model, this condition is incorporated by assuming that the system *S* and all the ancillas are in a product state, that is:

(4) $$\begin{array}{c} {\hat{\rho }}_{SE}\left(0\right)={\hat{\rho }}_{S}\left(0\right)\underset{i}{⨂}{\hat{\eta }}_{E_{i}}. \end{array}$$

We thus see that not only the system and the environment are in a product state, but also that there are no correlations between ancillas. It is possible to consider situations where there are correlations between ancillas, but this in general gives rise to non-Markovian dynamics, i.e., to memory effects.

Before moving on and describing how the dynamics is modeled in the microscopic and collisional approach, we need to discuss the form of the interaction Hamiltonian. In the microscopic approach one typically assumes that this is of the form:

(5) $$\begin{array}{c} {\hat{H}}_{SE}=\sum_{\alpha }{\hat{A}}_{S}^{\left(\alpha \right)}⊗{\hat{B}}_{E}^{\left(\alpha \right)} \end{array}$$

where ${\hat{A}}_{S}^{\left(\alpha \right)}$ and ${\hat{B}}_{E}^{\left(\alpha \right)}$ are system and environment operators, respectively, which can be assumed, without loss of generality, to be Hermitian. Moreover, in order to obtain a Markovian master equation, this interaction Hamiltonian is assumed to represent a small perturbation with respect to the free Hamiltonian ${\hat{H}}_{S}$, ${\hat{H}}_{E}$. In the collision model, one needs to specify the interaction Hamiltonian between *S* and all the ancillas $E_{i}$. For a time-independent Hamiltonian, like the one in Equation (5), one can simply assume that these interaction Hamiltonians are all isomorphic, i.e., they are all of the form:

(6) $$\begin{array}{c} {\hat{H}}_{SE_{i}}=g\sum_{\alpha }{\hat{A}}_{S}^{\left(\alpha \right)}⊗{\hat{B}}_{E_{i}}^{\left(\alpha \right)} \end{array}$$

Note also that in Equation (6) we introduced the constant *g* that gauges the strength of the interaction between *S* and the ancillas. This constant will be useful when expanding in power series to keep track of the order expansion.

### 2.2. Modeling the Dynamics

In the microscopic approach, in order to study the dynamics of the system, it is convenient to move to the interaction picture with respect to the free Hamiltonian ${\hat{H}}_{S}+{\hat{H}}_{E}$.

We thus define the density matrix ${\hat{\rho }}_{SE}$ and operators ${\hat{O}}_{SE}$ acting on the joint Hilbert space in the interaction picture as:

(7) $$\begin{array}{c} {\hat{\rho }}_{SE}^{I}=e^{i({\hat{H}}_{S}+{\hat{H}}_{E})t}{\hat{\rho }}_{SE}e^{-i({\hat{H}}_{S}+{\hat{H}}_{E})t}, \end{array}$$

(8) $$\begin{array}{c} {\hat{O}}_{SE}^{I}=e^{-i({\hat{H}}_{S}+{\hat{H}}_{E})t}{\hat{O}}_{SE}e^{+i({\hat{H}}_{S}+{\hat{H}}_{E})t}, \end{array}$$

where from now on we will drop the apex *I* and put it back only when confusion may arise. Note also that we have set ℏ=1.

In the interaction picture, one has that the unitary dynamics of the joint system is thus given by:

(9) $$\begin{array}{c} \frac{d{\hat{\rho }}_{SE}\left(t\right)}{dt}=-i\left[{\hat{H}}_{SE}\left(t\right),{\hat{\rho }}_{SE}\left(t\right)\right], \end{array}$$

which implies that the state of the joint system can be written as:

(10) $$\begin{array}{c} {\hat{\rho }}_{SE}\left(t\right)={\hat{\rho }}_{SE}\left(0\right)-i\int_{0}^{t}ds\left[{\hat{H}}_{SE}\left(s\right),{\hat{\rho }}_{SE}\left(s\right)\right]. \end{array}$$

Finally, one can insert Equation (10) into Equation (9) to obtain an integro-differential equation of second order in ${\hat{H}}_{SE}$, and after tracing away the environmental degrees of freedom one can write:

(11) $$\begin{array}{c} \frac{d{\hat{\rho }}_{S}\left(t\right)}{dt}=-\int_{0}^{t}ds\mathrm{Tr}\{\left[{\hat{H}}_{SE}\left(t\right),\;[{\hat{H}}_{SE}\left(s\right),{\hat{\rho }}_{SE}\left(s\right)]\right]\}, \end{array}$$

which represents the starting point of the derivation of the Markovian GKSL equation in the microscopic approach. In writing Equation (11), we assumed that the following stability condition holds, namely:

(12) $$\begin{array}{c} {\mathrm{Tr}}_{E}\{{\hat{H}}_{SE}{\hat{\rho }}_{SE}\left(0\right)\}=\mathrm{Tr}\left\{{\hat{B}}_{E}^{\left(\alpha \right)}{\hat{\rho }}_{E}\right\}=0. \end{array}$$

This is the only assumption made up to now, and we will see shortly that a similar assumption is needed also in the collisional approach. Anyway, it must be highlighted that this assumption implies no loss of generality, as it can always be enforced by appropriately rescaling the free Hamiltonian ${\hat{H}}_{S}$ [47]. Specifically, in the case $\mathrm{Tr}\left\{{\hat{B}}_{E}^{\left(\alpha \right)}{\hat{\rho }}_{E}\right\}=\langle {\hat{B}}_{E}^{\left(\alpha \right)}\rangle \neq 0$, one can define the displaced operator:

(13) $$\begin{array}{c} {\hat{B}}_{E}^{{\left(\alpha \right)}^{\prime }}={\hat{B}}_{E}^{\left(\alpha \right)}-\langle {\hat{B}}_{E}^{\left(\alpha \right)}\rangle \end{array}$$

Substituting this into the interaction Hamiltonian in Equation (5), one obtains:

(14) $$\begin{array}{c} {\hat{H}}_{SE}=\sum_{\alpha }{\hat{A}}_{S}^{\left(\alpha \right)}⊗{\hat{B}}_{E}^{{\left(\alpha \right)}^{\prime }}+\sum_{\alpha }\langle {\hat{B}}_{E}^{\left(\alpha \right)}\rangle {\hat{A}}_{S}^{\left(\alpha \right)}. \end{array}$$

One can immediately see that the second contribution corresponds to a renormalization of the system Hamiltonian ${\hat{H}}_{S}$, while the first contribution is a new interaction Hamiltonian, where now the stability condition in Equation (12) holds.

Apart from this stability condition, until this point no approximations or assumptions have been made yet, and Equation (11) is still exact. Notice also that Equation (11) is of second order with respect to the interaction Hamiltonian ${\hat{H}}_{SE}$.

**Figure 3:**
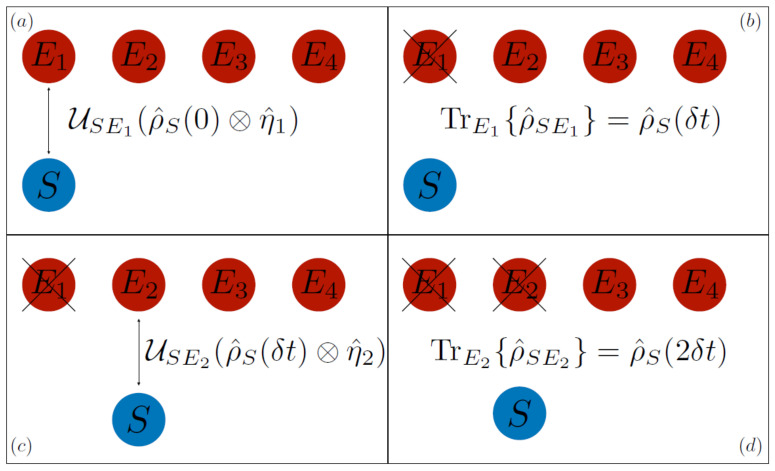
Schematic representation of the collisional dynamics in the Markovian case. In panel (**a**), the system interacts with the first ancilla $E_{1}$ through the unitary map $U_{SE_{1}}$. In panel (**b**), after the interaction has taken place, the ancilla $E_{1}$ is traced away, leaving us with the state of the system after the interaction ${\hat{\rho }}_{S}\left(\delta t\right)$. Then in panel (**c**), the system interacts with the second ancilla $E_{2}$ through the unitary map $U_{SE_{2}}$. Finally, in panel (**d**), the ancilla $E_{2}$ is traced away, leaving us with the state ${\hat{\rho }}_{S}\left(2\delta t\right)$. Repeating these two steps, interaction and tracing, for all the ancillas, gives rise to the open system dynamics of *S*.

Let us now derive the equivalent expression in the collisional approach, pictorially summarized in Figure 3. In this case, the system starts its dynamics by interacting with the first ancilla through the interaction Hamiltonian ${\hat{H}}_{SE_{1}}$ according to the unitary map $U_{SE_{1}}$:

(15) $$\begin{array}{c} {\hat{\rho }}_{S}\left(0\right)⊗{\hat{\eta }}_{E_{1}}\to U_{SE_{1}}\left({\hat{\rho }}_{S}\left(0\right)⊗{\hat{\eta }}_{E_{1}}\right)={\hat{U}}_{SE_{1}}{\hat{\rho }}_{S}\left(0\right)⊗{\hat{\eta }}_{E_{1}}{\hat{U}}_{SE_{1}}^{†} \end{array}$$

where the unitary operator ${\hat{U}}_{SE_{1}}$ stems from the unitary dynamics induced by the interaction Hamiltonian ${\hat{H}}_{SE_{1}}$:

(16) $$\begin{array}{c} {\hat{U}}_{SE_{1}}=\exp\left[-i{\hat{H}}_{SE_{1}}\delta t\right]. \end{array}$$

Note that, at glance with the microscopic derivation, in the collision model we do not move to the interaction picture.

The quantity δt is the collision time, i.e., the time during which the collision happens and the system interacts with the ancilla $E_{1}$. After the first collision, the degrees of freedom of the ancilla $E_{1}$ are traced away, leaving us with the state:

(17) $$\begin{array}{c} {\hat{\rho }}_{S}\left(\delta t\right)={\mathrm{Tr}}_{E_{1}}\left\{U_{SE_{1}}\left({\hat{\rho }}_{S}\left(0\right)⊗{\hat{\eta }}_{E_{1}}\right)\right\}. \end{array}$$

At this point, the system *S* interacts with the ancilla $E_{2}$ according to:

(18) $$\begin{array}{c} {\hat{\rho }}_{S}\left(\delta t\right)⊗{\hat{\eta }}_{E_{2}}\to U_{SE_{2}}\left({\hat{\rho }}_{S}\left(\delta t\right)⊗{\hat{\eta }}_{E_{2}}\right). \end{array}$$

Then, we once again trace away the environment, obtaining the state of the system after two collisions:

(19) $$\begin{array}{c} {\hat{\rho }}_{S}\left(2\delta t\right)=\mathrm{Tr}\left\{U_{SE_{2}}\left({\hat{\rho }}_{S}⊗{\hat{\eta }}_{E_{2}}\right)\right\}=\mathrm{Tr}\left\{U_{SE_{2}}\circ U_{SE_{1}}\left(\hat{\rho }\left(0\right)⊗{\hat{\eta }}_{E_{1}}⊗{\hat{\eta }}_{E_{2}}\right)\right\}. \end{array}$$

It is then easy to see that after the n-th collision, the state of the system *S* can be written as:

(20) $$\begin{array}{c} {\hat{\rho }}_{S}\left(n\delta t\right)={\mathrm{Tr}}_{E_{1},E_{2},\ldots ,E_{n}}\left\{U_{SE_{n}}\circ U_{SE_{n-1}}\circ \cdots \circ U_{SE_{1}}\left({\hat{\rho }}_{S}\left(0\right)\overset{n}{\underset{i_{1}}{⨂}}{\hat{\eta }}_{E_{i}}\right)\right\} \end{array}$$

In order to obtain the collisional equivalent of Equation (11) we need to write an expression of second order with respect to the interaction Hamiltonian ${\hat{H}}_{SE_{n}}$. In order to do so, we expand the operator ${\hat{U}}_{SE_{n}}$ in power series with respect to gδt, obtaining:

(21) $$\begin{array}{c} {\hat{U}}_{SE_{n}}=\hat{I}-ig\delta t{\hat{H}}_{SE_{n}}-\frac{{\left(g\delta t\right)}^{2}}{2}{\hat{H}}_{SE_{n}}^{2}+O({\left(g\delta t\right)}^{3}), \end{array}$$

so that the action of the unitary map $U_{SE_{n}}$ on the state $\hat{\rho }\left(\left(n-1\right)\delta t\right)⊗{\hat{\eta }}_{E_{n}}$ can be written as:

(22) $$\begin{array}{c} U_{SE_{n}}\left({\hat{\rho }}_{S}\left(\left(n-1\right)\delta t\right)⊗{\hat{\eta }}_{E_{n}}\right)\\ ={\hat{U}}_{SE_{n}}\left({\hat{\rho }}_{S}\left(\left(n-1\right)\delta t\right)⊗{\hat{\eta }}_{E_{n}}\right){\hat{U}}_{SE_{n}}^{†}\\ ={\hat{\rho }}_{S}\left(\left(n-1\right)\delta t\right)⊗{\hat{\eta }}_{E_{n}}-ig\delta t[{\hat{H}}_{SE_{n}},{\hat{\rho }}_{S}\left(\left(n-1\right)\delta t\right)⊗{\hat{\eta }}_{E_{n}}]\\ +{\left(g\delta t\right)}^{2}{\hat{H}}_{SE_{n}}\left({\hat{\rho }}_{S}\left(\left(n-1\right)\delta t\right)⊗{\hat{\eta }}_{E_{n}}\right){\hat{H}}_{SE_{n}}-\frac{{\left(g\delta t\right)}^{2}}{2}\{{\hat{H}}_{SE_{n}}^{2},{\hat{\rho }}_{S}\left(\left(n-1\right)\delta t\right)⊗{\hat{\eta }}_{E_{n}}\} \end{array}$$

Upon assuming the stability condition, which in the collisional approach reads:

(23) $$\begin{array}{c} {\mathrm{Tr}}_{E_{i}}\left\{{\hat{H}}_{SE_{i}}\left({\hat{\rho }}_{S}\left(\left(i-1\right)\delta t\right)⊗{\hat{\eta }}_{E_{i}}\right)\right\}=0\;\forall \;i, \end{array}$$

we can finally write, after tracing the ancilla degrees of freedom:

(24) $$\begin{array}{c} \frac{{\hat{\rho }}_{S}\left(n\delta t\right)-{\hat{\rho }}_{S}\left(\left(n-1\right)\delta t\right)}{\delta t}=\\ \;\;\;g^{2}\delta t{\mathrm{Tr}}_{E_{n}}\left\{2{\hat{H}}_{SE_{n}}\left({\hat{\rho }}_{S}⊗{\hat{\eta }}_{E_{n}}\right){\hat{H}}_{SE_{n}}-\frac{1}{2}\{{\hat{H}}_{SE_{n}},{\hat{\rho }}_{S}\left(\left(n-1\right)\delta t\right)⊗{\hat{\eta }}_{E_{n}}\}\right\}. \end{array}$$

Equation (25) is the collisional equivalent of Equation (11): in fact, on the lhs of the equation we find the difference between the state of the system at two different times, divided by the collision time δt, which is nothing but a discrete form of the derivative with respect to time appearing in Equation (11). On the rhs we find instead an expression which is of second order with respect to the interaction Hamiltonian ${\hat{H}}_{SE_{n}}$. It is also important to highlight that in performing the series expansion in Equation (21), we assumed for simplicity the interaction Hamiltonian is time independent, as otherwise another term would appear [45,52].

Up to now, it might seem that the only difference between the microscopic and collisional approaches is that one is time-continuous and the other discrete. In the next subsection, we are going to see how the Born and Markov approximations allow one to get deeper into the dynamics of the open system, and this will allow us also to see more significant differences between the two approaches.

### 2.3. The Born-Markov Approximation

Let us now go back to Equation (11): while this equation looks very elegant, it is not yet very informative, and is also hard to treat, as it is not clear how to deal with the huge number of environmental degrees of freedom. Thus, some approximations are required in order to simplify the expression.

The first of these approximations is the *Born approximation*: with this approximation, one assumes that, as the coupling is weak and the environment is very large, the interaction between *S* and E only slightly affects the state of the environment, so that the states of *S* and E are factorized at all times, i.e.,

(25) $$\begin{array}{c} {\hat{\rho }}_{SE}\left(t\right)≃{\hat{\rho }}_{S}\left(t\right)⊗{\hat{\rho }}_{E} \end{array}$$

It is important to stress that this approximation does not imply that the environment is not affected by the interaction with *S* or that no excitations are created in the environment, but only that these excitations decay much faster than the typical timescale at which the dynamics of *S* happens. We can already see that we do not need to make this approximation in the collisional approach, in the sense that the Born approximation is already embedded in the model, since all the ancillas are initially uncorrelated with *S*.

Inserting Equation (25) into Equation (11), we obtain:

(26) $$\begin{array}{c} \frac{d{\hat{\rho }}_{S}\left(t\right)}{dt}=-\int_{0}^{t}ds\mathrm{Tr}\{[{\hat{H}}_{SE}\left(t\right),\;[{\hat{H}}_{SE}\left(s\right),{\hat{\rho }}_{S}\left(s\right)⊗{\hat{\rho }}_{E}]]\}, \end{array}$$

which is one step further towards our goal, but still is not simple enough to allow for further calculations. In fact, the problem with Equation (26) is that it is non-local in time, i.e., it still contains the state of the system at a previous time *s*, and thus is non-Markovian. Thus, in order to derive a Markovian master equation, we need to enforce the *Markov approximation*, which consists in substituting ${\hat{\rho }}_{S}\left(s\right)$ with ${\hat{\rho }}_{S}\left(t\right)$, obtaining the *Redfield equation*:

(27) $$\begin{array}{c} \frac{d{\hat{\rho }}_{S}\left(t\right)}{dt}=-\int_{0}^{t}ds\mathrm{Tr}\{[{\hat{H}}_{SE}\left(t\right),\;[{\hat{H}}_{SE}\left(s\right),{\hat{\rho }}_{S}\left(t\right)⊗{\hat{\rho }}_{E}]]\}. \end{array}$$

At a glance with the microscopic approach, the Markov approximation is already embedded in the collision model, and thus need not be enforced in Equation (25): this feature is due to both the fact that the ancillas are all initially uncorrelated, i.e., in a product state, and also to the fact that after the interaction with the system *S* the ancilla is immediately traced away.

In order to guarantee the positivity of Equation (27), we need one further assumption, namely that the relaxation time $\tau_{R}$ at which the state of the joint system changes appreciably is much larger than the timescale $\tau_{E}$ at which the environmental correlation functions, to be defined in the next subsection, decay (see also [53,54]). If this is the case, then it is possible to make the substitution s→t−s in Equation (27) and let the integration limit go to infinity:

(28) $$\begin{array}{c} \frac{d{\hat{\rho }}_{S}\left(t\right)}{dt}=-\int_{0}^{\infty }ds\mathrm{Tr}\{[{\hat{H}}_{SE}\left(t\right),\;[{\hat{H}}_{SE}\left(t-s\right),{\hat{\rho }}_{S}\left(t\right)⊗{\hat{\rho }}_{E}]]\}. \end{array}$$

### 2.4. The Secular Approximation

We are almost ready to write the Markovian master equation in the GKSL form. To do this, we need to write the interaction Hamiltonian ${\hat{H}}_{SE}$ in Equation (5) in terms of the eigenoperators of ${\hat{H}}_{S}$. This can be done by considering the projectors ${\hat{\Pi }}_{\epsilon }$ onto the subspace with energy ϵ and defining:

(29) $$\begin{array}{c} {\hat{A}}_{S}^{\left(\alpha \right)}\left(\omega \right)=\sum_{\epsilon -\epsilon^{\prime }=\omega }{\hat{\Pi }}_{\epsilon^{\prime }}{\hat{A}}_{S}^{\left(\alpha \right)}{\hat{\Pi }}_{\epsilon } \end{array}$$

so that the interaction Hamiltonian becomes:

(30) $$\begin{array}{c} {\hat{H}}_{SE}=\sum_{\alpha ,\omega }{\hat{A}}_{S}^{\left(\alpha \right)}\left(\omega \right)⊗{\hat{B}}_{E}^{\left(\alpha \right)}. \end{array}$$

The advantage of writing the interaction Hamiltonian in terms of the eigenoperators of ${\hat{H}}_{S}$ is that this allows us to write it in the interaction picture in a very simple fashion:

(31) $$\begin{array}{c} {\hat{H}}_{SE}\left(t\right)=\sum_{\alpha ,\omega }e^{-i\omega t}{\hat{A}}_{S}^{\left(\alpha \right)}\left(\omega \right)⊗{\hat{B}}_{E}^{\left(\alpha \right)}\left(t\right) \end{array}$$

As one can see, thanks to the decomposition in terms of eigenoperators of ${\hat{H}}_{S}$, the time evolution of the interation Hamiltonian simply reduces to a phase. We can substitute Equation (31) into Equation (28) and develop the commutator, obtaining:

(32) $$\begin{array}{ccc} \frac{d{\hat{\rho }}_{S}\left(t\right)}{dt} & = & \int_{0}^{\infty }ds{\mathrm{Tr}}_{E}\{{\hat{H}}_{SE}\left(t-s\right)\left({\hat{\rho }}_{S}\left(t\right)⊗{\hat{\rho }}_{E}\right){\hat{H}}_{SE}\left(t\right)-\left({\hat{\rho }}_{S}\left(t\right)⊗{\hat{\rho }}_{E}\right){\hat{H}}_{SE}\left(t-s\right){\hat{H}}_{SE}\left(t\right)\\ & + & {\hat{H}}_{SE}\left(t\right)\left({\hat{\rho }}_{S}\left(t\right)⊗{\hat{\rho }}_{E}\right){\hat{H}}_{SE}\left(t-s\right)-{\hat{H}}_{SE}\left(t\right){\hat{H}}_{SE}\left(t-s\right)\left({\hat{\rho }}_{S}\left(t\right)⊗{\hat{\rho }}_{E}\right)\}\\ & = & \sum_{\begin{array}{c} \omega ,\omega^{\prime }\\ \alpha ,\beta \end{array}}e^{i(\omega^{\prime }-\omega )t}\int_{0}^{\infty }ds\;e^{i\omega s}{\mathrm{Tr}}_{E}\left\{{\hat{B}}_{E}^{(\alpha )†}\left(t\right){\hat{B}}_{E}^{\left(\beta \right)}\left(t-s\right){\hat{\rho }}_{E}\right\}[{\hat{A}}_{S}^{\left(\beta \right)}\left(\omega^{\prime }\right){\hat{\rho }}_{S}{\hat{A}}_{S}^{(\alpha )†}\left(\omega \right)\\ & - & {\hat{A}}_{S}^{(\alpha )†}\left(\omega^{\prime }\right){\hat{A}}_{S}^{\left(\beta \right)}\left(\omega \right){\hat{\rho }}_{S}\left(t\right)+{\hat{A}}_{S}^{(\alpha )†}\left(\omega \right){\hat{\rho }}_{S}{\hat{A}}_{S}^{\left(\beta \right)}\left(\omega^{\prime }\right)-{\hat{\rho }}_{S}\left(t\right){\hat{A}}_{S}^{\left(\beta \right)}\left(\omega \right){\hat{A}}_{S}^{(\alpha )†}\left(\omega^{\prime }\right)]\; \end{array}$$

The last equation can be written in a more concise fashion by defining the Fourier transform of the environmental two-time correlation functions as:

(33) $$\begin{array}{c} \Gamma_{\alpha \beta }\left(\omega \right)=\int ds\;e^{i\omega s}{\mathrm{Tr}}_{E}\left\{{\hat{B}}_{E}^{(\alpha )†}\left(t\right){\hat{B}}_{E}^{\left(\beta \right)}\left(t-s\right){\hat{\rho }}_{E}\right\} \end{array}$$

where it has been assumed that ${\hat{\rho }}_{E}$ is a stationary state of the environment, i.e., that $[{\hat{H}}_{E}{\hat{\rho }}_{E}]=0$, so that $\Gamma_{\alpha \beta }\left(\omega \right)$ is time-independent. With this definition, Equation (33) can be written as:

(34) $$\begin{array}{ccc} \frac{d{\hat{\rho }}_{S}\left(t\right)}{dt} & = & \sum_{\begin{array}{c} \omega ,\omega^{\prime }\\ \alpha ,\beta \end{array}}\Gamma_{\alpha \beta }\left(\omega \right)e^{i(\omega^{\prime }-\omega )t}[{\hat{A}}_{S}^{\left(\beta \right)}\left(\omega^{\prime }\right){\hat{\rho }}_{S}{\hat{A}}_{S}^{(\alpha )†}\left(\omega \right)-{\hat{A}}_{S}^{(\alpha )†}\left(\omega^{\prime }\right){\hat{A}}_{S}^{\left(\beta \right)}\left(\omega \right){\hat{\rho }}_{S}\left(t\right)\\ & & \;\;+{\hat{A}}_{S}^{(\alpha )†}\left(\omega \right){\hat{\rho }}_{S}\left(t\right){\hat{A}}_{S}^{\left(\beta \right)}\left(\omega^{\prime }\right)-{\hat{\rho }}_{S}\left(t\right){\hat{A}}_{S}^{\left(\beta \right)}\left(\omega \right){\hat{A}}_{S}^{(\alpha )†}\left(\omega^{\prime }\right)] \end{array}$$

Let us now take a closer look at Equation (34), where we have eigenoperators relative to different eigenfrequencies $\omega ,\omega^{\prime }$. The dynamics of *S* happens at a timescale $\tau_{S}≃{\left|\omega -\omega^{\prime }\right|}^{-1}$; that is, $\tau_{S}$ is the timescale at which the state of *S* changes appreciably. If this timescale is much smaller than the timescale $\tau_{R}$ at which the joint S+E system evolves, then it is possible to neglect the terms with $\omega \neq \omega^{\prime }$ in Equation (34). This is the so-called *secular approximation*, which allows us to write:

(35) $$\begin{array}{ccc} \frac{d{\hat{\rho }}_{S}\left(t\right)}{dt} & = & \sum_{\begin{array}{c} \omega \\ \alpha ,\beta \end{array}}\Gamma_{\alpha \beta }\left(\omega \right)[{\hat{A}}_{S}^{\left(\beta \right)}\left(\omega \right){\hat{\rho }}_{S}{\hat{A}}_{S}^{(\alpha )†}\left(\omega \right)-{\hat{A}}_{S}^{(\alpha )†}\left(\omega \right){\hat{A}}_{S}^{\left(\beta \right)}\left(\omega \right){\hat{\rho }}_{S}\left(t\right)\\ & & \;\;+{\hat{A}}_{S}^{(\alpha )†}\left(\omega \right){\hat{\rho }}_{S}{\hat{A}}_{S}^{\left(\beta \right)}\left(\omega \right)-{\hat{\rho }}_{S}\left(t\right){\hat{A}}_{S}^{\left(\beta \right)}\left(\omega \right){\hat{A}}_{S}^{(\alpha )†}\left(\omega \right)] \end{array}$$

Thanks to the secular approximation, we have a much simpler expression, as now only terms where the operators have the same frequency ω appear.

Let us now go back to the collisional approach. Substituting Equation (6) into Equation (25), we obtain immediately:

(36) $$\begin{array}{c} \frac{{\hat{\rho }}_{S}\left(n\delta t\right)-{\hat{\rho }}_{S}\left(\left(n-1\right)\delta t\right)}{\delta t}=\\ g^{2}\delta t\sum_{\alpha ,\beta }\Gamma_{\alpha \beta }\left({\hat{A}}_{S}^{\left(\alpha \right)}{\hat{\rho }}_{S}\left(\left(n-1\right)\delta t\right){\hat{A}}_{S}^{(\beta )†}-\frac{1}{2}\{{\hat{A}}_{S}^{(\beta )†}{\hat{A}}_{S}^{\left(\alpha \right)},{\hat{\rho }}_{S}\left(\left(n-1\right)\delta t\right)\}\right). \end{array}$$

where in this case we define the environment correlation functions as:

(37) $$\begin{array}{c} \Gamma_{\alpha \beta }={\mathrm{Tr}}_{E_{n}}\left\{{\hat{B}}_{E_{n}}^{(\beta )†}{\hat{B}}_{E_{n}}^{\left(\alpha \right)}{\hat{\eta }}_{E_{n}}\right\}. \end{array}$$

It is worth noticing at this point some differences with the microscopic approach. First, we did not need to express the interaction Hamiltonian ${\hat{H}}_{SE_{n}}$ in terms of eigenoperators of ${\hat{H}}_{S}$. This feature is once again due to the discrete nature of the collision model, while in the microscopic approach, having depicted the environment as a continuum of modes, we needed to select the collective modes actually interacting with the system *S*.

In second place, we did not need to invoke the secular approximation: while this feature might still look as if it is due to the discreteness of the model, it is actually due to the fact that each collision between system and ancillas is by construction described by a completely positive and trace-preserving (CPT) map. This has important and deep consequences: first of all the positivity of Equation (36) is already guaranteed, since a combination of CPT maps is still a CPT map. This is to be compared with the microscopic approach, where the secular approximation is a necessary condition to obtain complete positivity. Furthermore, dynamical evolution described by a combination of CPT maps implies the possibility of writing a master equation in Lindblad form [6,51].

At this point, only one more step is needed to derive the semigroup generator in GKSL form, as we are going to see in the next subsection.

### 2.5. The GKSL Generator

In order to write the final form of the GKSL master equation, we need to manipulate the matrix $\Gamma_{\alpha \beta }$. This manipulation is basically the same in both approaches, apart from the different definition. In fact, the matrix $\Gamma_{\alpha \beta }\left(\omega \right)$ can be written as:

(38) $$\begin{array}{ccc} \Gamma_{\alpha \beta }\left(\omega \right) & = & \frac{1}{2}\Gamma_{\alpha \beta }\left(\omega \right)+\frac{1}{2}\Gamma_{\alpha \beta }\left(\omega \right)=\frac{1}{2}\left(\Gamma_{\alpha \beta }\left(\omega \right)+\Gamma_{\beta \alpha }^{*}\left(\omega \right)\right)+\frac{i}{2}\left(-i\Gamma_{\alpha \beta }\left(\omega \right)+i\Gamma_{\beta \alpha }^{*}\left(\omega \right)\right)\\ & = & \frac{1}{2}\gamma_{\alpha \beta }\left(\omega \right)+i\Sigma_{\alpha \beta }\left(\omega \right) \end{array}$$

where we have defined

(39) $$\begin{array}{ccc} \gamma_{\alpha \beta }\left(\omega \right) & = & \Gamma_{\alpha \beta }\left(\omega \right)+\Gamma_{\beta \alpha }^{*}\left(\omega \right), \end{array}$$

(40) $$\begin{array}{ccc} \Sigma_{\alpha \beta }\left(\omega \right) & = & -\frac{i}{2}\left(\Gamma_{\alpha \beta }\left(\omega \right)-\Gamma_{\beta \alpha }^{*}\left(\omega \right)\right). \end{array}$$

The matrix $\Sigma_{\alpha \beta }$ is Hermitian for fixed ω, while the matrix $\gamma_{\alpha \beta }\left(\omega \right)$ is positive. Every positive matrix can be diagonalized through a unitary operator $u_{\alpha \beta }\left(\omega \right)$, so that:

(41) $$\begin{array}{c} \gamma_{\alpha \beta }\left(\omega \right)=\sum_{\gamma }u_{\alpha \gamma }\left(\omega \right)\gamma_{\gamma }^{\prime }u_{\gamma \beta }^{†}\left(\omega \right) \end{array}$$

where the matrix $\gamma_{\gamma }^{\prime }\left(\omega \right)$ is a real diagonal matrix. Through the unitary $u_{\alpha \beta }\left(\omega \right)$, it is also possible to define the new operators

(42) $$\begin{array}{c} {\hat{A}}_{S}^{{}^{\prime }\left(\alpha \right)}\left(\omega \right)=\sum_{\beta }u_{\alpha \beta }{\hat{A}}_{S}^{\left(\beta \right)}\left(\omega \right), \end{array}$$

which are linear combinations of the original operators ${\hat{A}}_{S}^{\left(\alpha \right)}\left(\omega \right)$.

Using the definitions in Equations (39), (40), (42) we can rewrite Equation (35) as:

(43) $$\begin{array}{ccc} \frac{d{\hat{\rho }}_{S}\left(t\right)}{dt} & = & -i[{\hat{H}}_{LS},{\hat{\rho }}_{S}\left(t\right)]\\ & + & \sum_{\omega ,\alpha }\gamma_{\alpha }^{\prime }\left(\omega \right)\left({\hat{A}}_{S}^{{}^{\prime }\left(\alpha \right)}\left(\omega \right){\hat{\rho }}_{S}{\hat{A}}_{S}^{{}^{\prime }\left(\alpha \right)†}\left(\omega \right)-\frac{1}{2}\left\{{\hat{A}}_{S}^{{}^{\prime }\left(\alpha \right)†}\left(\omega \right){\hat{A}}_{S}^{{}^{\prime }\left(\alpha \right)}\left(\omega \right),{\hat{\rho }}_{S}\left(t\right)\right\}\right) \end{array}$$

where the Hermitian operator ${\hat{H}}_{LS}\left(t\right)$ is known as the *Lamb shift* and is defined as:

(44) $$\begin{array}{c} {\hat{H}}_{LS}=\sum_{\begin{array}{c} \omega \\ \alpha ,\beta \end{array}}\Sigma_{\alpha \beta }\left(\omega \right){\hat{A}}_{S}^{(\alpha )†}\left(\omega \right){\hat{A}}_{S}^{\left(\beta \right)}\left(\omega \right). \end{array}$$

Equation (43) is the GKSL form of the master equation, and the operators ${\hat{A}}_{S}^{{}^{\prime }\left(\alpha \right)}$ are called *Lindblad operators*: it contains a unitary part of the dynamics, dictated by the commutator term, and a dissipative part, dictated by the sum of the terms on the second line.

With the same procedure, we can write the final GKSL form of the master equation in the collisional approach, obtaining:

(45) $$\begin{array}{c} \frac{{\hat{\rho }}_{S}\left(n\delta t\right)-{\hat{\rho }}_{S}\left(\left(n-1\right)\delta t\right)}{\delta t}=-i[{\hat{H}}_{LS},{\hat{\rho }}_{S}\left(\left(n-1\right)\delta t\right)]\\ +g^{2}\delta t\sum_{\omega ,\alpha }\gamma_{\alpha }^{\prime }\left(\omega \right)\left({\hat{A}}_{S}^{{}^{\prime }\left(\alpha \right)}\left(\omega \right){\hat{\rho }}_{S}\left(\left(n-1\right)\delta t\right){\hat{A}}_{S}^{{}^{\prime }\left(\alpha \right)†}\left(\omega \right)-\frac{1}{2}\{{\hat{A}}_{S}^{{}^{\prime }\left(\alpha \right)†}\left(\omega \right){\hat{A}}_{S}^{{}^{\prime }\left(\alpha \right)}\left(\omega \right),{\hat{\rho }}_{S}\left(\left(n-1\right)\delta t\right)\}\right). \end{array}$$

We see that the main difference between Equations (43) and (45) is the discreteness of the time parameter: we are going to see in the next subsection how it is possible to transform Equation (45) into a time-continuous equation.

We are also at a good point to present the main differences between the collisional and microscopic approaches, as presented in Table 1.

The first assumption we made was the stability condition, as in Equations (12) and (23): these two equations look very similar indeed, and the only difference between the two is given by the fact that the microscopic model is time-continuous while the collision model has a discrete fashion.

An important difference between the two approaches has been met first when dealing with the Born and Markov approximations. In the microscopic approach, both approximations had to be enforced through Equations (25) and (27), respectively. In the collision model, on the other hand, there was no need to enforce these approximations, as they are already included in the initial conditions of the model. Namely, the Born approximation is given, in the collisional approach, by the fact that the initial state of the ancillas is a tensor product state with the state of the environment, and as the interaction is always between the system *S* and a single ancilla $E_{i}$, and as the ancilla degrees of freedom are traced away immediately afterwards, no correlations are retained in the system. As for the Markov approximations, since there are no intra-ancilla interactions, i.e., collisions between different ancillas, no memory effects are present: in fact, collision models giving rise to non-Markovian dynamics are often obtained by allowing for such intra-ancilla interactions, so that partial memory of the previous collisions is retained, though memory effects can be introduced also by other means [28].

Finally, the last approximation we enforced in the microscopic approach was the secular approximation. This approximation involves the ratio between the typical timescale of the system, defined by $|\omega -\omega^{\prime }{|}^{-1}$, and the typical relaxation time $\tau_{R}$ of the joint system, and it is enforced in order to guarantee the complete positivity of the dynamical evolution. Opposite to this, in the collision model the complete positivity is guaranteed by the fact that each collision between the system *S* and one of the ancillas is completely positive by definition, and thus the combination of CPT maps is also completely positive. This is one of the most distinctive feature of collision models, and one of the main reasons they allow for a simple derivation of the master equation.

### 2.6. The Continuous Time Limit

In this subsection, we briefly explain how to enforce the continuous time limit in the collision model. While at first sight this might seem strange, as the collision model is inherently discrete, the continuous time limit can lead to a continuous time master equation directly from a simple discrete model. Once again, this is to be compared with the microscopic approach, where one needs to enforce a series of approximations, thus making more complicated the derivation of the final result.

To enforce this limit, one needs at the same time to make the number of collisions and the interaction strength infinite, while making the collision time infinitesimal, so as to have:

(46) $$\begin{array}{c} \lim_{\begin{array}{c} g\to \infty \\ \delta t\to 0 \end{array}}g^{2}\delta t=\gamma \end{array}$$

Within this limit, one has that the product between the number of collisions and the collision time becomes the time variable, while the collision time becomes a differential:

(47) $$\begin{array}{c} \lim_{\begin{array}{c} \delta t\to 0\\ n\to \infty \end{array}}n\delta t=t \end{array}$$

Inserting all this limit into Equation (45), one has that the lhs of the equation becomes:

(48) $$\begin{array}{c} \lim_{\begin{array}{c} \delta t\to 0\\ n\to \infty \end{array}}\frac{{\hat{\rho }}_{S}\left(n\delta t\right)-{\hat{\rho }}_{S}\left(\left(n-1\right)\delta t\right)}{\delta t}=\frac{d{\hat{\rho }}_{S}\left(t\right)}{dt}, \end{array}$$

while the rhs of Equation (45) becomes:

(49) $$\begin{array}{c} \lim_{\delta t\to 0}\lim_{n\to \infty }\lim_{g\to \infty }-i[{\hat{H}}_{LS},{\hat{\rho }}_{S}\left(\left(n-1\right)\delta t)\right]\\ +g^{2}\delta t\sum_{\omega ,\alpha }\gamma_{\alpha }^{\prime }\left(\omega \right)\left({\hat{A}}_{S}^{{}^{\prime }\left(\alpha \right)}\left(\omega \right){\hat{\rho }}_{S}\left(\left(n-1\right)\delta t\right){\hat{A}}_{S}^{{}^{\prime }\left(\alpha \right)†}\left(\omega \right)-\frac{1}{2}\{{\hat{A}}_{S}^{{}^{\prime }\left(\alpha \right)†}\left(\omega \right){\hat{A}}_{S}^{{}^{\prime }\left(\alpha \right)}\left(\omega \right),{\hat{\rho }}_{S}\left(\left(n-1\right)\delta t\right)\}\right)\\ =-i[{\hat{H}}_{LS},{\hat{\rho }}_{S}\left(t\right)]+\gamma \sum_{\omega ,\alpha }\gamma_{\alpha }^{\prime }\left(\omega \right)\left({\hat{A}}_{S}^{{}^{\prime }\left(\alpha \right)}\left(\omega \right){\hat{\rho }}_{S}\left(t\right){\hat{A}}_{S}^{{}^{\prime }\left(\alpha \right)†}\left(\omega \right)-\frac{1}{2}\{{\hat{A}}_{S}^{{}^{\prime }\left(\alpha \right)†}\left(\omega \right){\hat{A}}_{S}^{{}^{\prime }\left(\alpha \right)}\left(\omega \right),{\hat{\rho }}_{S}\left(t\right)\}\right). \end{array}$$

Thus one can see that after the continuous time limit has been enforced, Equation (50) becomes basically identical to Equation (43). It must be noted, however, that much care is needed when enforcing such a limit, as there are situations in which it can give rise to pathological situations, see [51] for an extensive discussion of this issue.

## 3. Examples

In order to better understand the collisional approach, we are now going to examine some very simple examples of two-level systems (TLS) and harmonic oscillators interacting with thermal baths. Such scenarios are among the most simple and common that one can find, and thus very instructive without unnecessary complications stemming from more articulated physical systems.

### 3.1. TLS Interacting with a Bosonic Bath

As a first example, we want to consider a TLS, characterized by the two levels |*e*〉, |*g*〉, with free Hamiltonian

(50) $$\begin{array}{c} {\hat{H}}_{S}=\frac{1}{2}\hbar \omega_{0}{\hat{\sigma }}_{z} \end{array}$$

where ${\hat{\sigma }}_{z}$ is the usual Pauli matrix defined as:

(51) $$\begin{array}{c} {\hat{\sigma }}_{z}=|e\rangle \langle e|-|g\rangle \langle g|=\left[\begin{array}{cc} 1 & 0\\ 0 & -1 \end{array}\right]. \end{array}$$

This TLS interacts with a polarized monochromatic electric field in a thermal state, i.e., a bosonic mode. We thus model the environment as a set of ancillas $\left\{E_{i}\right\}$ characterized by the Hamiltonian

(52) $$\begin{array}{c} {\hat{H}}_{E_{i}}=\hbar \omega {\hat{b}}_{E_{i}}^{†}{\hat{b}}_{E_{i}} \end{array}$$

where ${\hat{b}}_{E_{i}}^{†},\;{\hat{b}}_{E_{i}}$ are bosonic creation and annihilation operators, respectively, fulfilling the commutation relations:

(53) $$\begin{array}{c} {}[{\hat{b}}_{E_{i}}{\hat{b}}_{E_{j}}^{†}]=\delta_{ij}. \end{array}$$

Moreover, we assume that the frequency $\omega ≃\omega_{0}$, so that each ancilla is almost at resonance with the frequency of the TLS.

As we want to study the effect of the interaction with a thermal field, we write the state of the ancillas as:

(54) $$\begin{array}{c} {\hat{\eta }}_{E_{i}}=\frac{e^{-\beta {\hat{H}}_{E_{i}}}}{Z_{E_{i}}},\;Z_{E_{i}}=\mathrm{Tr}\left\{e^{-\beta {\hat{H}}_{E_{i}}}\right\}={\left(1-e^{-\beta \hbar \omega }\right)}^{-1}, \end{array}$$

where $\beta ={\left(k_{B}T\right)}^{-1}$ is the inverse temperature and $Z_{E_{i}}$ is the so-called partition function.

To a first approximation, the interaction between the TLS and the field is given by the dipole interaction. In cases of light polarized along the *x* direction, the dipole interaction between the TLS and the electric field can be written as:

(55) $$\begin{array}{c} {\hat{H}}_{SE_{i}}=g\left({\hat{\sigma }}_{+}{\hat{b}}_{E_{i}}+{\hat{\sigma }}_{-}{\hat{b}}_{E_{i}}^{†}\right), \end{array}$$

where *g* is a constant containing all the information about the dipole moment matrix element and the interaction strength, while the operators ${\hat{\sigma }}_{\pm }$ are defined as:

(56) $$\begin{array}{c} {\hat{\sigma }}_{+}=|e\rangle \langle g|=\left[\begin{array}{cc} 0 & 1\\ 0 & 0 \end{array}\right], \end{array}$$

(57) $$\begin{array}{c} {\hat{\sigma }}_{-}=|g\rangle \langle e|=\left[\begin{array}{cc} 0 & 0\\ 1 & 0 \end{array}\right]. \end{array}$$

The first thing to notice is that the stability condition is fulfilled, since:

(58) $$\begin{array}{c} {\mathrm{Tr}}_{E_{i}}\left\{{\hat{H}}_{SE_{i}}\left({\hat{\rho }}_{S}\left(i-1\right)⊗{\hat{\eta }}_{E_{i}}\right)\right\}=\frac{g}{Z_{E_{i}}}{\mathrm{Tr}}_{E_{i}}\left\{\left({\hat{\sigma }}_{+}{\hat{b}}_{E_{i}}+{\hat{\sigma }}_{-}{\hat{b}}_{E_{i}}^{†}\right)\left({\hat{\rho }}_{S}\left(0\right)⊗e^{-\beta \hbar \omega {\hat{b}}_{E_{i}}^{†}{\hat{b}}_{E_{i}}}\right)\right\}=0. \end{array}$$

At this point we can immediately compute the environmental correlation functions, which in this case are given by:

(59) $$\begin{array}{c} \gamma_{{\hat{b}}^{†}\hat{b}}=\mathrm{Tr}\left\{{\hat{b}}^{†}\hat{b}\frac{e^{-\beta \hbar \omega {\hat{b}}^{†}\hat{b}}}{Z}\right\}=N_{\beta }={\left(e^{\beta \hbar \omega }-1\right)}^{-1}, \end{array}$$

(60) $$\begin{array}{ccc} & & \gamma_{\hat{b}{\hat{b}}^{†}}=\mathrm{Tr}\left\{\hat{b}{\hat{b}}^{†}\frac{e^{-\beta \hbar \omega {\hat{b}}^{†}\hat{b}}}{Z}\right\}=N_{\beta }+1={\left(e^{\beta \hbar \omega }-1\right)}^{-1}+1, \end{array}$$

(61) $$\begin{array}{ccc} & & \gamma_{\hat{b}\hat{b}}=0,\;\gamma_{{\hat{b}}^{†}{\hat{b}}^{†}}=0. \end{array}$$

We are then ready to write the master equation as:

(62) $$\begin{array}{ccc} {\hat{\rho }}_{S}\left(n+1\right)={\hat{\rho }}_{S}\left(n\right) & + & {\left(g\delta t\right)}^{2}\{\left(N_{\beta }+1\right)\left[{\hat{\sigma }}_{-}{\hat{\rho }}_{S}\left(n\right){\hat{\sigma }}_{+}-\frac{1}{2}\{{\hat{\sigma }}_{+}{\hat{\sigma }}_{-},{\hat{\rho }}_{S}\left(n\right)\}\right]\\ & + & N_{\beta }\left[{\hat{\sigma }}_{+}{\hat{\rho }}_{S}\left(n\right){\hat{\sigma }}_{-}-\frac{1}{2}\{{\hat{\sigma }}_{-}{\hat{\sigma }}_{+},{\hat{\rho }}_{S}\left(n\right)\}\right]\}. \end{array}$$

As one can see, thanks to the simplicity of the collision model, we have been able to write down the master equation after just a few lines of calculations.

It is easy to find the steady state ${\hat{\rho }}_{S}^{\mathrm{steady}}$ of Equation (62) by imposing the condition ${\hat{\rho }}_{S}\left(n+1\right)={\hat{\rho }}_{S}\left(n\right)$, which implies:

(63) $$\begin{array}{c} \left(N_{\beta }+1\right)\left[{\hat{\sigma }}_{-}{\hat{\rho }}_{S}^{\mathrm{steady}}{\hat{\sigma }}_{+}-\frac{1}{2}\{{\hat{\sigma }}_{+}{\hat{\sigma }}_{-},{\hat{\rho }}_{S}^{\mathrm{steady}}\}\right]+N_{\beta }\left[{\hat{\sigma }}_{+}{\hat{\rho }}_{S}^{\mathrm{steady}}{\hat{\sigma }}_{-}-\frac{1}{2}\{{\hat{\sigma }}_{-}{\hat{\sigma }}_{+},{\hat{\rho }}_{S}^{\mathrm{steady}}\}\right]=0\\ \Rightarrow -N_{\beta }\left[\begin{array}{cc} \left(\rho_{11}^{\mathrm{steady}}-\rho_{22}^{\mathrm{steady}}\right) & \rho_{12}^{\mathrm{steady}}\\ \rho_{21}^{\mathrm{steady}} & -(\rho_{11}^{\mathrm{steady}}-\rho_{22}^{\mathrm{steady}}) \end{array}\right]-\left[\begin{array}{cc} \rho_{11}^{\mathrm{steady}} & \rho_{12}^{\mathrm{steady}}/2\\ \rho_{21}^{\mathrm{steady}}/2 & -\rho_{11}^{\mathrm{steady}} \end{array}\right]=0. \end{array}$$

Using Equation (63) together with the condition $\rho_{11}^{\mathrm{steady}}+\rho_{22}^{\mathrm{steady}}=1$, we obtain:

(64) $$\begin{array}{c} \rho_{11}^{\mathrm{steady}}=\frac{1}{e^{\beta \hbar \omega_{0}}+1} \end{array}$$

(65) $$\begin{array}{c} \rho_{22}^{\mathrm{steady}}=\frac{e^{\beta \hbar \omega_{0}}}{e^{\beta \hbar \omega_{0}}+1}, \end{array}$$

(66) $$\begin{array}{c} \rho_{12}^{\mathrm{steady}}=0. \end{array}$$

These matrix elements are nothing but those of a thermal state at temperature β, i.e.,

(67) $$\begin{array}{c} {\hat{\rho }}_{S}^{\mathrm{steady}}=\frac{e^{-\beta \hbar \omega_{0}\hat{\sigma_{z}}}}{\mathrm{Tr}\{e^{-\beta \hbar \omega_{0}\hat{\sigma_{z}}}\}}. \end{array}$$

It is important to note also that the detailed balance condition is satisfied since:

(68) $$\begin{array}{c} \frac{\rho_{11}^{\mathrm{steady}}}{\rho_{22}^{\mathrm{steady}}}=e^{-\beta \hbar \omega_{0}}. \end{array}$$

### 3.2. Bosonic Mode Interacting with a Bosonic Bath

As a second example, we want to consider a bosonic mode interacting with a bosonic bath. At a glance, with the previous example, the system Hamiltonian is now given by:

(69) $$\begin{array}{c} {\hat{H}}_{S}=\hbar \omega_{0}{\hat{a}}^{†}\hat{a}, \end{array}$$

where ${\hat{a}}^{†},\hat{a}$ are the creation and annihilation operators for the bosonic mode, fulfilling the standard commutation relation $[\hat{a},{\hat{a}}^{†}]=\hat{I}$.

As in the previous example, we model the environment as a set of ancillas $\left\{E_{i}\right\}$ in the thermal state ${\hat{\eta }}_{E_{i}}$ characterized by the Hamiltonian in Equation (52).

Since now the system is described by the operators $\hat{a},{\hat{a}}^{†}$, we write the interaction Hamiltonian between the system and each ancilla in the dipole approximation as:

(70) $$\begin{array}{c} {\hat{H}}_{SE_{i}}=g\left(\hat{a}{\hat{b}}_{E_{i}}^{†}+{\hat{a}}^{†}{\hat{b}}_{E_{i}}\right) \end{array}$$

As the environmental correlation functions are identical to the ones from the previous section, it is immediately apparent that the master equation can be written as follows:

(71) $$\begin{array}{ccc} {\hat{\rho }}_{S}\left(n+1\right)={\hat{\rho }}_{S}\left(n\right) & + & {\left(g\delta t\right)}^{2}\{\left(N_{\beta }+1\right)\left[\hat{a}{\hat{\rho }}_{S}\left(n\right){\hat{a}}^{†}-\frac{1}{2}\{{\hat{a}}^{†}\hat{a},{\hat{\rho }}_{S}\left(n\right)\}\right]\\ & + & N_{\beta }\left[{\hat{a}}^{†}{\hat{\rho }}_{S}\left(n\right)\hat{a}-\frac{1}{2}\{\hat{a}{\hat{a}}^{†},{\hat{\rho }}_{S}\left(n\right)\}\right]\}. \end{array}$$

As can be seen, Equation (71) is very similar to the master equation in Equation (62), with the only difference being that the operators ${\hat{\sigma }}_{+},{\hat{\sigma }}_{-}$ have been substituted by the operators ${\hat{a}}^{†},\hat{a}$, respectively.

Using Equation (71), it is possible to compute the steady state of the expectation value of any combination of operators $\hat{a},{\hat{a}}^{†}$. In particular, one can find the steady state populations by imposing the condition:

(72) $$\begin{array}{c} \mathrm{Tr}\left\{{\hat{a}}^{†}\hat{a}\left({\hat{\rho }}_{S}\left(n+1\right)-{\hat{\rho }}_{S}\left(n\right)\right)\right\}=0. \end{array}$$

From this condition, inserting Equation (71) and exploiting the commutation relations of the operators $\hat{a},{\hat{a}}^{†}$, one obtains:

(73) $$\begin{array}{ccc} & & {\left(g\delta t\right)}^{2}\left(N_{\beta }+1\right)\mathrm{Tr}\left\{{\hat{a}}^{†}\hat{a}\hat{a}{\hat{\rho }}_{S}\left(n\right){\hat{a}}^{†}-{\hat{a}}^{†}\hat{a}{\hat{a}}^{†}\hat{a}{\hat{\rho }}_{S}\left(n\right)\right\}\\ & & +{\left(g\delta t\right)}^{2}N_{\beta }\mathrm{Tr}\left\{{\hat{a}}^{†}\hat{a}{\hat{a}}^{†}{\hat{\rho }}_{S}\left(n\right)\hat{a}-\frac{1}{2}{\hat{a}}^{†}\hat{a}\hat{a}{\hat{a}}^{†}{\hat{\rho }}_{S}\left(n\right)-\frac{1}{2}{\hat{a}}^{†}\hat{a}{\hat{\rho }}_{S}\left(n\right)\hat{a}{\hat{a}}^{†}\right\}=0\\ \Rightarrow & & {\left(g\delta t\right)}^{2}\left[-\left(N_{\beta }+1\right)\mathrm{Tr}\left\{{\hat{a}}^{†}\hat{a}{\hat{\rho }}_{S}\left(n\right)\right\}+N_{\beta }\left(\mathrm{Tr}\left\{{\hat{a}}^{†}\hat{a}{\hat{\rho }}_{S}\left(n\right)\right\}+1\right)\right]=0\\ \Rightarrow & & \mathrm{Tr}\left\{{\hat{a}}^{†}\hat{a}{\hat{\rho }}_{S}\left(n\right)\right\}=N_{\beta }. \end{array}$$

As in the previous example, the populations become thermal, which implies that the system thermalizes at the temperature β of the environment. This is perfectly analogous to the case of a TLS interacting with the bosonic bath, with the main difference being the fact that we exploited the algebra of the operators $\hat{a},{\hat{a}}^{†}$ to perform the calculations.

### 3.3. TLS Interacting with a TLS Bath

The last example we consider is once again a TLS, but this time interacting with a bath composed by TLSs. Thus, the Hamiltonian of the system is the same as in Equation (50), while we model the environment as a set of ancillas $\left\{E_{i}\right\}$ with Hamiltonian

(74) $$\begin{array}{c} {\hat{H}}_{E_{i}}=\frac{1}{2}\hbar \omega {\hat{\sigma }}_{E_{i}}^{z}, \end{array}$$

that is, each ancilla is a TLS in the state ${\hat{\eta }}_{E_{i}}$ that for the moment does not need to be specified.

We can write the most general interaction between two TLSs as:

(75) $$\begin{array}{c} {\hat{H}}_{SE_{i}}=g_{ex}\left({\hat{\sigma }}_{S}^{+}{\hat{\sigma }}_{E_{i}}^{-}+{\hat{\sigma }}_{S}^{-}{\hat{\sigma }}_{E_{i}}^{+}\right)+g_{dec}{\hat{\sigma }}_{S}^{z}{\hat{\sigma }}_{E_{i}}^{z}. \end{array}$$

The first part of this interaction Hamiltonian is responsible for the exchange of excitations between *S* and the ancilla $E_{i}$, and thus is equivalent to the interaction Hamiltonian in Equation (55) with the operators ${\hat{\sigma }}^{\pm }$ substituting $\hat{b},{\hat{b}}^{†}$. The term proportional to $g_{dec}$ is instead responsible for pure decoherence, as we are going to see explicitly.

The first thing to do is to verify whether the stability condition holds. In order to do this, we need to compute the following traces:

(76) $$\begin{array}{c} \mathrm{Tr}\left\{{\hat{\sigma }}_{E_{i}}^{+}\hat{\eta }\right\}=\eta_{21},\;\mathrm{Tr}\left\{{\hat{\sigma }}_{E_{i}}^{-}\hat{\eta }\right\}=\eta_{12},\;\mathrm{Tr}\left\{{\hat{\sigma }}_{E_{i}}^{z}\hat{\eta }\right\}=\eta_{11}-\eta_{22} \end{array}$$

where we have parametrized the ancilla state ${\hat{\eta }}_{E_{i}}$ as:

(77) $$\begin{array}{c} {\hat{\eta }}_{E_{i}}=\left[\begin{array}{cc} \eta_{11} & \eta_{12}\\ \eta_{21} & \eta_{22} \end{array}\right] \end{array}$$

We note that for the stability condition to hold one surely needs $\eta_{12}=\eta_{21}^{*}=0$, i.e., the environmental state must not have any coherence. This is actually a more general fact, since the first moments of creation and annihilation (fermionic or bosonic) are typically zero for diagonal states such as thermal states or the vacuum.

We also notice that the third term in Equation (76) is never zero, apart from the case of the maximally mixed state. This is anyway not a problem, because the corresponding term in the master equation is proportional to ${\hat{\sigma }}_{S}^{z}$, and thus to the Hamiltonian ${\hat{H}}_{S}$, so that we can eliminate it through an appropriate renormalization of the frequency of the TLS. This is an example of how the stability condition can be always enforced, as anticipated in Section 2. We will thus neglect this term in what follows.

Assuming that the ancilla state is diagonal (i.e., $\eta_{12}=\eta_{21}^{*}=0$), one can compute the environmental correlation function as:

(78) $$\begin{array}{ccc} & & {\hat{\Gamma }}_{{\hat{\sigma }}^{+}{\hat{\sigma }}^{-}}={\mathrm{Tr}}_{E_{i}}\left\{{\hat{\sigma }}^{+}{\hat{\sigma }}^{-}{\hat{\eta }}_{E_{i}}\right\}=\eta_{11} \end{array}$$

(79) $$\begin{array}{ccc} & & {\hat{\Gamma }}_{{\hat{\sigma }}^{-}{\hat{\sigma }}^{+}}={\mathrm{Tr}}_{E_{i}}\left\{{\hat{\sigma }}^{-}{\hat{\sigma }}^{+}{\hat{\eta }}_{E_{i}}\right\}=\eta_{22} \end{array}$$

(80) $$\begin{array}{ccc} & & {\hat{\Gamma }}_{{\hat{\sigma }}^{z}{\hat{\sigma }}^{z}}={\mathrm{Tr}}_{E_{i}}\left\{{\hat{\sigma }}^{z}{\hat{\sigma }}^{z}{\hat{\eta }}_{E_{i}}\right\}=1, \end{array}$$

so that the master equation finally reads:

(81) $$\begin{array}{ccc} \frac{{\hat{\rho }}_{S}\left(n+1\right)-{\hat{\rho }}_{S}\left(n\right)}{\delta t} & = & g_{ex}^{2}\delta t\;\eta_{11}\left[{\hat{\sigma }}_{S}^{-}{\hat{\rho }}_{S}\left(n\right){\hat{\sigma }}_{S}^{+}-\frac{1}{2}\{{\hat{\sigma }}_{S}^{+}{\hat{\sigma }}_{S}^{-},{\hat{\rho }}_{S}\left(n\right)\}\right]\\ & + & g_{ex}^{2}\delta t\;\eta_{22}\left[{\hat{\sigma }}_{S}^{+}{\hat{\rho }}_{S}\left(n\right){\hat{\sigma }}_{S}^{-}-\frac{1}{2}\{{\hat{\sigma }}_{S}^{-}{\hat{\sigma }}_{S}^{+},{\hat{\rho }}_{S}\left(n\right)\}\right]\\ & + & g_{dec}^{2}\delta t\left[{\hat{\sigma }}_{S}^{z}{\hat{\rho }}_{S}\left(n\right){\hat{\sigma }}_{S}^{z}-{\hat{\rho }}_{S}\left(n\right)\right] \end{array}$$

where in the last row we have exploited the identity ${\hat{\sigma }}_{S}^{z}{\hat{\sigma }}_{S}^{z}=I$.

We can now analyze the effect of each row of Equation (81). The first two rows, analogously to Equation (62), are responsible for the exchange of excitation into and from the bath, respectively. In particular, one can notice that, in agreement with the physics of fermions, the first row, which described the exchange of excitations from the system into the environment, is proportional to the population of the ground state of the environment: if $\eta_{11}=0$, this would mean that all the spin in the environment is in their excited state, and thus that there would not be any space for an excitation coming from the system.

Conversely, the second row is responsible for the absorption of excitations from the environment into the system: if $\eta_{22}=0$, this would mean that environment were in its ground state, and thus that there would be no excitations that the system could absorb.

Finally, the last term of Equation (81) has no analogue in Equation (62): we can easily verify that this term is responsible for pure decoherence, i.e., decoherence without dissipation. To see this we compute explicitly the effect of this term. We have that:

(82) $$\begin{array}{c} {\hat{\sigma }}_{S}^{z}{\hat{\rho }}_{S}\left(n\right){\hat{\sigma }}_{S}^{z}=\left[\begin{array}{cc} 1 & 0\\ 0 & -1 \end{array}\right]\left[\begin{array}{cc} \rho_{11}\left(n\right) & \rho_{12}\left(n\right)\\ \rho_{21}\left(n\right) & \rho_{22}\left(n\right) \end{array}\right]\left[\begin{array}{cc} 1 & 0\\ 0 & -1 \end{array}\right]=\left[\begin{array}{cc} \rho_{11}\left(n\right) & -\rho_{12}\left(n\right)\\ -\rho_{21}\left(n\right) & \rho_{22}\left(n\right) \end{array}\right] \end{array}$$

so that one has that the density matrix at the n+1 step can be written as:

(83) $$\begin{array}{c} \left[\begin{array}{cc} \rho_{11}\left(n+1\right) & \rho_{12}\left(n+1\right)\\ \rho_{21}\left(n+1\right) & \rho_{22}\left(n+1\right) \end{array}\right]=\left[\begin{array}{cc} \rho_{11}\left(n\right) & \rho_{12}\left(n\right)\\ \rho_{21}\left(n\right) & \rho_{22}\left(n\right) \end{array}\right]-2{\left(g_{dec}\delta t\right)}^{2}\left[\begin{array}{cc} 0 & \rho_{12}\left(n\right)\\ \rho_{21}\left(n\right) & 0 \end{array}\right]. \end{array}$$

From Equation (83), we can see that the effect of the Lindblad term with the operator ${\hat{\sigma }}_{S}^{z}$ has the effect of reducing the off-diagonal terms of the density matrix, that is, the coherence of the state, thus driving any initial state into a purely diagonal state, without affecting the population.

This, together with the other two terms of Equation (81), implies that the steady state of the system is a state without coherence (i.e., diagonal) whose populations are the same as the one of the environment. This means that we are dealing with a homogenizing process, in which the system is driven step by step towards the same state as the environment.

## 4. Conclusions

In this tutorial we have examined how collision models work by deriving the Markovian GKSL generator. This was done step by step, highlighting the approximations made and their meaning with respect to physical systems. Moreover, the collisional approach was compared step by step with its microscopic counterpart, thus highlighting the differences between the two approaches, in order to better explain the strengths and weaknesses of the two approaches.

We have also shown how to practically use the concept illustrated in Section 2 via three paradigmatic examples, namely a quantum TLS interacting with either a bosonic or a TLS bath, and the case of a bosonic system interacting with a bosonic bath.

We hope that this tutorial can be useful to anybody interested in starting using collision models, as they represent a useful tool in the study of the open quantum system and there is a large community of researchers working with them.

## Figures and Tables

**Table 1:** Summary of the main differences between the microscopic and collisional approaches. The stability condition is very similar in the two approaches, and the main difference is given only by the discrete nature of the collision model. The Born and Markov approximations are instead where the differences between the two approaches become the most evident: in facts, while in the microscopic approach one needs to enforce both during the derivation of the master equation, in the collisional approach these two approximations are already encompassed in the initial conditions, namely the fact that all the ancillas do not share any initial correlation and that they do not interact with each other during the dynamics. As a matter of fact, non-Markovianity is usually introduced in collision models by allowing interactions between the ancillas so that some memory effect is retained. Finally, also the secular approximation is identical in both approaches, as it deals with the ratio between the typical timescale of the system *S* and the one of the joint system. However, as highlighted in the main text, the secular approximation is usually not needed in most collision models, as the interaction Hamiltonian is chosen so as to conserve the particle number.

Approximation	Microscopic Approach	Collisional Approach
Stability condition	${\mathrm{Tr}}_{E}\{{\hat{H}}_{SE}{\hat{\rho }}_{SE}\left(0\right)\}=0$	${\mathrm{Tr}}_{E_{i}}\{{\hat{H}}_{SE_{i}}\left({\hat{\rho }}_{S}\left(\left(i-1\right)\delta t\right)⊗{\hat{\eta }}_{E_{i}}\right)\}=0$
Born approximation	${\hat{\rho }}_{SE}\left(t\right)≃{\hat{\rho }}_{S}\left(t\right)⊗{\hat{\eta }}_{E}$	Not needed, as it is already encompassed by the assumption of tensorized ancillas
Markov approximation	${\hat{\rho }}_{S}\left(s\right)\to {\hat{\rho }}_{S}\left(t\right)$	Not needed, as the ancillas do not interact with each other
Secular approximation	$\tau_{S}≃{\left|\omega -\omega^{\prime }\right|}^{-1}<<\tau_{R}$	Not needed, as each collision is already completely positive
